# Preparation, Characterization and Adsorption Potential of Grainy Halloysite-CNT Composites for Anthracene Removal from Aqueous Solution

**DOI:** 10.3390/nano9060890

**Published:** 2019-06-17

**Authors:** Gabriela Kamińska, Mariusz Dudziak, Edyta Kudlek, Jolanta Bohdziewicz

**Affiliations:** Institute of Water and Wastewater Engineering, Silesian University of Technology, Konarskiego 18, 44-100 Gliwice, Poland; mariusz.dudziak@polsl.pl (M.D.); edyta.kudlek@polsl.pl (E.K.); Jolanta.bohdziewicz@polsl.pl (J.B.)

**Keywords:** nanohalloysite, carbon nanotubes, adsorption, micropollutants

## Abstract

Grainy Hal-CNT composites were prepared from powder halloysite nanoclay (Hal) and carbon nanotubes (CNTs). The effect of the amount and type of CNTs, as well as calcination temperature on morphology and properties of Hal-CNT composites and their adsorption capacity of anthracene (ANT), were studied. The surface topography of granules was heterogenous, with cracks and channels created during granulation of powder clay and CNTs. In FTIR, spectra were exhibited only in the bands arising from halloysite, due to its dominance in the granules. The increase in the heating temperature to 550 °C resulted in mesoporosity/macroporosity of the granules, the lowest specific surface area (SSA) and poorest adsorption potential. Overall, SSA of all Hal-CNT composites were higher than raw Hal, and by itself, heated halloysite. The larger amount of CNTs enhanced adsorption kinetics due to the more external adsorption sites. The equilibrium was established with the contact time of approximately 30 min for the sample Hal-SWCNT 85:15, while the samples with loading 96:4, it was 60–90 min. Adsorption isotherms for ANT showed L1 type, which is representative for the sorbents with limited adsorption capacity. The Langmuir model described the adsorption process, suggesting a monolayer covering. The sample Hal-SWCNT 85:15 exhibited the highest adsorption capacity of ANT, due to its highest SSA and microporous character.

## 1. Introduction

Anthracene (ANT) is organic compound belonging to polycyclic aromatic hydrocarbons (PAHs) and has been identified by European Parliament in Water Framework Directive as particularly dangerous and a priority substance due to its carcinogenic and teratogenic effects and potential for bioaccumulation. The maximum allowable concentration of anthracene established by this directive is 0.1 µg/L for surface waters [1]. PAHs occur in aquatic environment as a consequence of: (1) Exploitation and utilization of petroleum; (2) incomplete combustion of fossil fuels; and (3) biogenic natural sources. They migrate into water sources by a deposition or direct discharge of fuel and oil products. PAHs contamination is present worldwide in sea water and surface water [2,3,4]. Accumulated PAHs in water sediments are considered as a re-emission source and pose additional concern due to the hazardous health effect when contaminating drinking water sources [5]. Therefore, it is important to reduce the risk related to PAHs occurrence in water by implementing efficient methods in water treatment technologies.

Adsorption has been found as the simplest and most economical process for the removal of PAHs, endocrine disrupting compounds, pesticides, and pharmaceuticals from water matrices [6,7]. Various adsorbents have been used for the removal of organic micro-pollutants including common activated carbon, clays, carbon nanotubes (CNTs), mesoporous silica, biomass wastes, and resin [8]. Each of the adsorbents have some specific features that make them the most appropriate to remove some groups of pollutants. Activated carbon, clays, and biomass waste are considered as low-cost adsorbents, while carbon nanotubes are very expensive [9]. On the other hand, carbon nanotubes exhibit several unique physical and chemical properties, such as large surface areas, and mechanical strength. It makes them good candidates for the removal of a wide group of pollutants with different properties from water and wastewater [10]. However, there are several obstacles resulting in their non-use in full-scale water and wastewater treatment plants. Firstly, when added to water in powder form, CNTs aggregate spontaneously due to very strong Van der Waals and hydrophobic interactions occurring along the axis of the tubes [11,12]. This caused a locking of some surface area and consequently, a decrease in adsorption capacity of CNTs [13]. Gogotsi and Presser [14] reported that theoretically, the specific surface area (SSA) of CNTs can reach 2600 m^2^/g, but due to tangling and aggregation, the SSA of CNTs is even 20 times lower. Another major concern of powder CNTs is there is no option of simple collection, regeneration and reuse of exhausted CNTs [15,16]. One strategy for regeneration of powder CNTs is chemical treatment, but this method generates waste solvents and causes undesirable chemical decomposition of adsorbates and their release to the cleaning solution [17,18].

Therefore, many efforts have been made to develop based material for carbon nanotubes in the easily separable and regenerable form. One approach includes binding of carbon nanotubes with the membrane or filter surface. These materials have been found mainly in the application of membrane processes where high or low pressure is required, generating additional expense [19,20,21]. Carbon nanotubes have been also fabricated in grainy form as hybrid or composite. Wei et al. [22] prepared a granular CNTs/pseudo-boehmite adsorbent using sol-gel method. The adsorbent exhibited high adsorption capacity for organic micro-pollutants and was easily thermally regenerated and reusable. Similarly, Mohammed and Baytak [23] synthesized bentonite-carbon nanotubes composite for the removal of rhodamide dye from water. Alternatively, as based material can also be used, other clay minerals including kaolinite, zeolite, montmorillonite [24,25] can also be used. All these clay minerals are relatively cheap, eco-friendly and nontoxic and were found to be efficient for the removal of metals, dyes, radionuclides and organic pollutants [26,27]. Another clay mineral having promising properties for adsorption is nanosized halloysite Al_2_(OH)_4_Si_2_O_5_·nH_2_O, belonging to the kaolin group. Halloysite (Hal) occurs naturally in the form of wrapped multilayer tubes. It exhibits chemical similarity to kaolinite, but each single layer in halloysite is separated by a monolayer of water [28]. Anastopoulos et al. [29] emphasized that, due to higher surface area, porosity and chemical composition, nanosized halloysite has better properties than non-porous micronsized kaolinite. Moreover, halloysite has a higher potential to be modified because of its internal and external surface. Owing to its mesoporous or even macroporous interior, halloysite finds a number of applications as a nanoscale container for polymers, a carrier for the loading and controlled release of guest molecules, adsorbent of pollution and a nanoreactor/nanotemplate for the synthesis of functional materials [30,31]. In spite of the high variety of possible applications of halloysite, the currently available literature data do not present its use as based material for carbon nanotubes. Halloysite has gained attention since the last decade when its unique properties and superiority over kaolinite were revealed. Previously, both were considered as poorer adsorbent compared to other clay minerals with high cation exchangeable capacity [29]. 

The objective of this study was to develop a novel granular halloysite-carbon nanotubes composite (Hal-CNT) with good adsorption capacity for organic micro-pollutants. For this purpose, this study used commercial halloysite, single walled carbon nanotubes (SWCNT) and single walled carbon nanotubes functionalized with hydroxyl groups (SWCNT-OH). Generally, six types of Hal-CNT composites were prepared varying in the type of carbon nanotubes, Hal/CNT ratio and thermal treatment conditions. Importantly, all sorbents appeared in the grainy form extending the number of applications, especially in context of water and wastewater treatment technologies. Anthracene was selected as adsorbate to represent the hydrophobic micro-pollutants in water matrices.

## 2. Materials and Methods

### 2.1. Materials

Nanohalloysite (Hal) powder was obtained from Sigma Aldrich (Poznań, Poland). Single walled carbon nanotubes (SWCNT) and single walled carbon nanotubes functionalized with hydroxyl group (SWCNT-OH) were obtained from Chengdu Organic Chemistry Co. Ltd. (Chengdu, China). The characteristics of the nanotubes are shown in Table 1. Deionized water was taken directly from Milli-Q water purification system (Millipore Merck, Warszawa, Poland). Acetonitrile (ACN), methanol (MeOH), dichloromethane (DMF) were purchased from Avantor Performance Materials (Gliwice, Poland). Anthracene was purchased from Sigma Aldrich (Poznań, Poland). Its physico-chemical properties are presented in Table 2. The stock solution of anthracene was prepared with acetonitrile (1 g/L). Then, a feed solution (C_0_ = 1 mg/L) was obtained by adding a sufficient volume of ANT stock solution to deionized water. 

### 2.2. Hal-CNT Composite Preparation

Grainy Hal-CNT composites were prepared from nanohalloysite powder and two different types of carbon nanotubes, i.e., SWCNT and SWCNT-OH. These two types of carbon nanotubes differed in the SSA and OH group. In our previous studies on adsorption on carbon nanotubes, it was found that their adsorption potential is not always clearly related to the SSA, and some other interactions (Van der Walls, hydrogen bonding, electrostatic interactions) can unexpectedly enhance adsorption making the sorbent more proper [7]. Therefore, the authors produced and studied individual granules containing SWCNT and SWCNT-OH. According to the modified method previously presented in [22,27], halloysite powder was added to carbon nanotubes with a weight ratio of 96:4 or 85:15. Following this, 1% nitric acid solution containing surfactant Brij (4 g/L) was added with a solid to liquid ratio of 30 g:10 mL and mixed for 30 min. Then, from the grease, cylindrical grains were formed with the diameter of 2 mm and a length 5 mm. The granules were heated for 60 min at 250 °C, followed by calcination (without air) in a kiln at 350 °C, 450 °C or 550 °C for 120 min. In addition, halloysite itself (without carbon nanotubes) was thermally treated according to the same procedure as Hal-CNT granules. Overall, nine different samples were prepared under various conditions, precisely listed in Table 3.

### 2.3. Halloysite-CNT Composite Characterization 

SEM/EDX images of Hal-CNT composites were obtained using a scanning electron microscope (SEM) NOVA NANO SEM 200 (Fei Europe Company, Hillsboro, OR, USA). Infrared spectra of raw materials (halloysite, SWCNT, SWCNT-OH) and selected Hal-CNT composites (i.e., Hal-SWCNT 85:15 and Hal-SWCNT-OH 85:15) were recorded with an infrared spectrometer Thermo Nicolet iS10 (Thermo Fisher Scientific, Waltham, MA, USA) equipped with ART cell. Spectra in the range of 4000–525 cm^−1^ were collected by 128 scans with a resolution of 4 cm^−1^. Nitrogen adsorption-desorption isotherms of all samples were estimated at 77 K by means of a volumetric adsorption analyzer ASAP 2010 (Micromeritics, Norcross, GA, USA) and then using a density functional theory (DFT) calculation method, the BET surface area and porous structure parameters were determined. 

### 2.4. Kinetic and Adsorption Study

The kinetic experiment was carried out adding 0.25 mg Hal-CNT samples to 100 mL of anthracene solution ($C_{0}=1\;\mathrm{mg}/L$) in glass flasks. The flasks were shaken at 200 rpm/min for 7 h under ambient conditions (20 ± 1 °C). At predetermined time intervals, the Hal-CNT samples were separated from the liquid using filter paper.

Similarly, an equilibrium adsorption experiment was conducted. However in this study, the initial concentration of ANT was changed in the range of 0.5–2 mg/L and the mass of the Hal-CNT sorbents varied from 0.1 mg to 4.2 mg. The flasks were shaken at 200 rpm/min to the moment where they gained equilibrium. Then, granules of Hal-CNT were separated from the anthracene solution by using filter paper.

All experiments were repeated twice. The blank sample (without adsorbent) was prepared to determine the losses of anthracene in the control sample. Additionally, kinetic and adsorption experiments were conducted for raw halloysite (Hal) and heated halloysite (Hal-350, Hal-450, Hal-550) was determined. 

The absorbed amount of ANT mined by means of Equation (1).

(1) $$Q_{e}=\frac{\left(C_{0}-C_{e}\right)*V}{m}$$

where: $Q_{e}$ (mg/g) is the equilibrium adsorption amount, $C_{0}$ and $C_{e}$ (mg/L) are the initial and equilibrium concentration of ANT, *m* (g) is the mass of sorbent, and *V* (L) is the volume of the solution. The concentration of ANT was measured with HPLC equipped with a chromatography column (Hypersil Gold C18, 5 µm particle size, 205 mm × 4.6 mm) and the UV-VIS detector at 254 nm. The flow rate of the mobile phase (acetonitrile/deionized water, 95/5, *v*/*v*) through the column was 1 mL/min. Before analysis, anthracene was extracted from the liquid by means of solid phase extraction (SPE) (phase C18, 1 g, 6 mL, Supelco, Sigma Aldrich, Poznań, Poland), according to previously developed methods [32].

## 3. Results

### 3.1. Characterization of Hal-CNT Composites 

**Figure 1:**
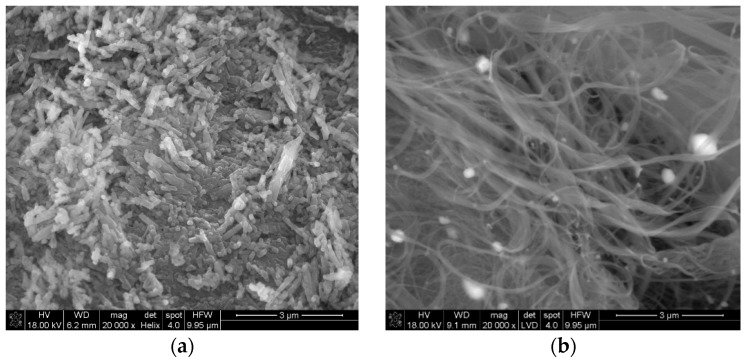
The SEM images of raw halloysite (**a**) and SWCNT (**b**).

The SEM images of raw Hal, SWCNT are presented in Figure 1. Hal demonstrated a relatively regular tubular shape, while SWCNT were tangled, due to a very strong interaction between individual tubes [33,34]. 

**Figure 2:**
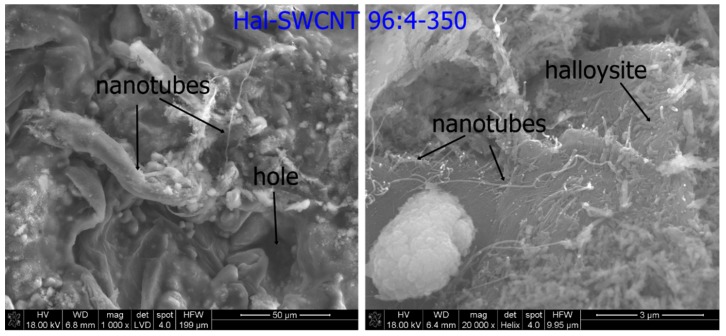

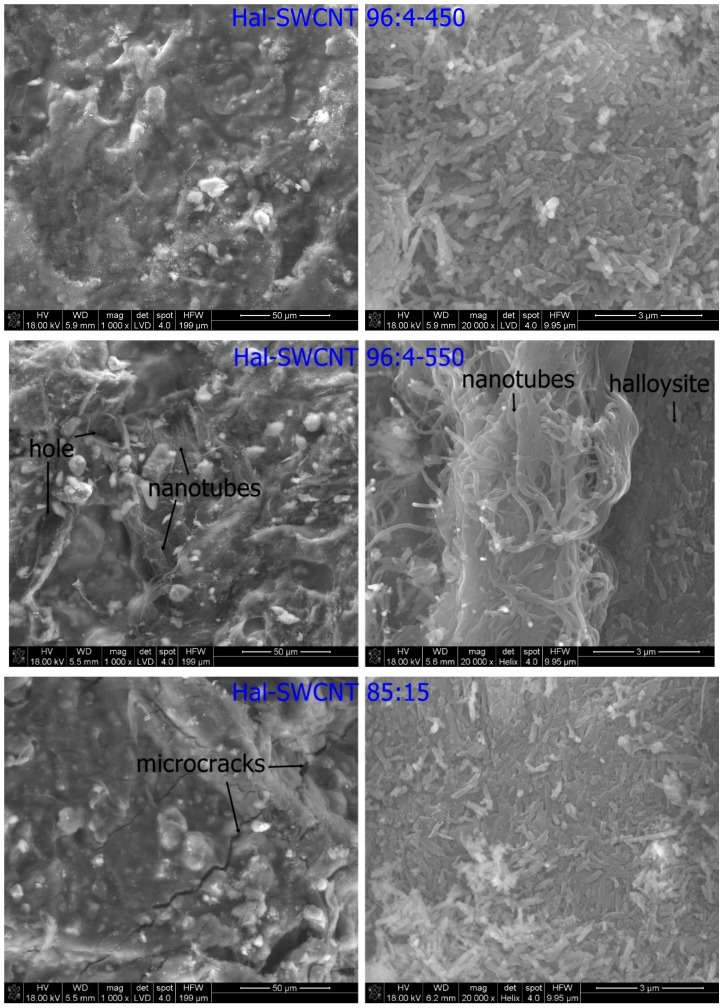

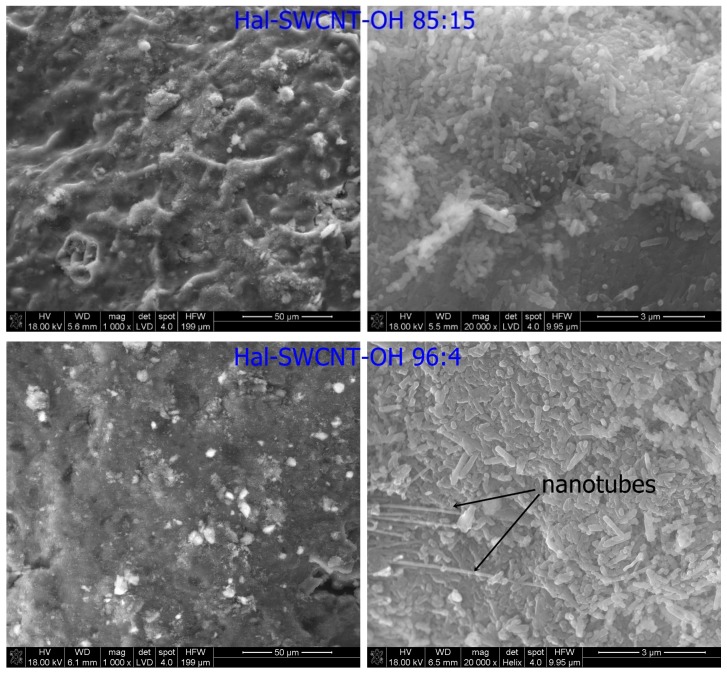
The SEM images of Hal-CNT composites.

The Halloysite tube was shorter and thicker than separate SWCNT or SWCNT-OH, thus they were well distinguishable in the Hal-CNT composites (Figure 2). 

The surface of granules was heterogeneous, rough with numerous clods, cracks and channels (Figure 2, left). When the granulation process occurs between particles of raw material, cracks are created. These cracks give a seedbed for mesopores in granulated powders that can enhance adsorption due to capillary condensation [35]. A digital photograph of granules was presented in Figure S1.

The calcination temperature had a negligible effect on the surface topography of the granules. In case of granules heated at 350 °C, 400 °C and 550 °C, halloysite kept a tubular shape with particle sizes of approximately 500 nm in length and 50–100 nm in diameter. Similarly, many authors observed no changes in the halloysite shape upon calcination [36,37]. In some parts of the granules, separate or tangled carbon nanotubes lied on the halloysite layer. There are three possible locations of carbon nanotubes in halloysite: (1) Internal lumen surface; (2) external surface; and (3) interlayer surface of halloysite [38]. It can be assumed that shorter carbon nanotubes entered into the halloysite internal lumen, while tangles of carbon nanotubes due to steric hindrance embedded on the surface.

**Figure 3:**
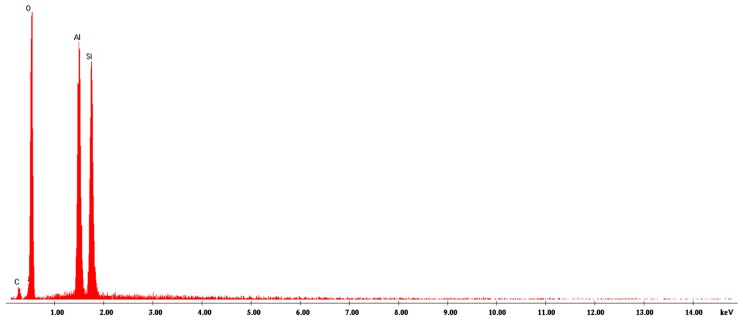
The EDX result of Hal-SWCNT 96:4-350.

The EDX (energy-dispersive X-ray) analysis indicates that the main composition of the Hal-CNT composites was C, O, Si, Al (Figure 3 and Figure S2). There were no peaks from other elements to indicate high purity of the product.

**Figure 4:**
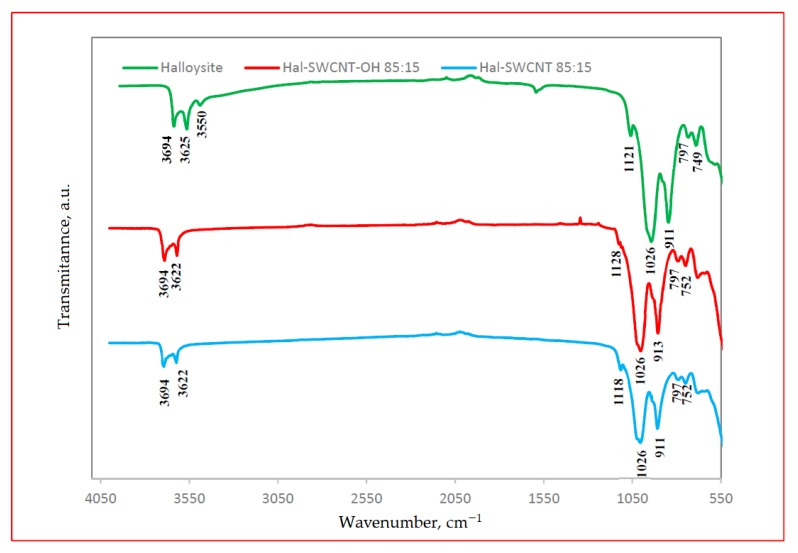
ATR-FTIR spectra of Hal, Hal-SWCNT 85:15, Hal-SWCNT-OH 85:15.

The FT-IR spectra of halloysite and Hal-SWCNT 85:15 and Hal-SWCNT-OH 85:15 are presented in Figure 4. The strong absorption band located for all samples at 3694–3622 cm^−1^ represented stretching vibrations of Al-OH of natural halloysite [39]. The spectral bands at 1026 cm^−1^ and 911 cm^−1^ arose from O-H deformation vibration of inner Al-OH groups [31]. The band at 797 cm^−1^ and in the region of 749–752 cm^−1^ were assigned to O-H translation vibrations of halloysite O-H components. The band of approximately 1121 cm^−1^ was attributed to Si-O stretching vibrations.

It was found that the FT-IR spectra of Hal-SWCNT 85:15 and Hal-SWCNT-OH 85:15 were dominated by bands typical for natural halloysite. Bands typical for raw SWCNT and SWCNT-OH (Figure S3) did not appear in the studied samples, probably due to high loading of halloysite. However, some differences between the spectra of raw halloysite and both Hal-CNT composites were found. In the raw halloysite spectra, there was a band at 3550 cm^−1^ corresponding to hydroxyl bands, which was reduced in Hal-SWCNT 85:15 and Hal-SWCNT-OH 85:15, probably due to dehydroxylation during calcination [40]. Secondly, the Si-O stretching band that appeared at 1121 cm^−1^ for halloysite was shifted for both composites to approximately 1118 cm^−1^. It can be explained by formation of hydrogen bonding between SWCNT or SWCNT-OH and the outer surface of halloysite.

**Figure 5:**
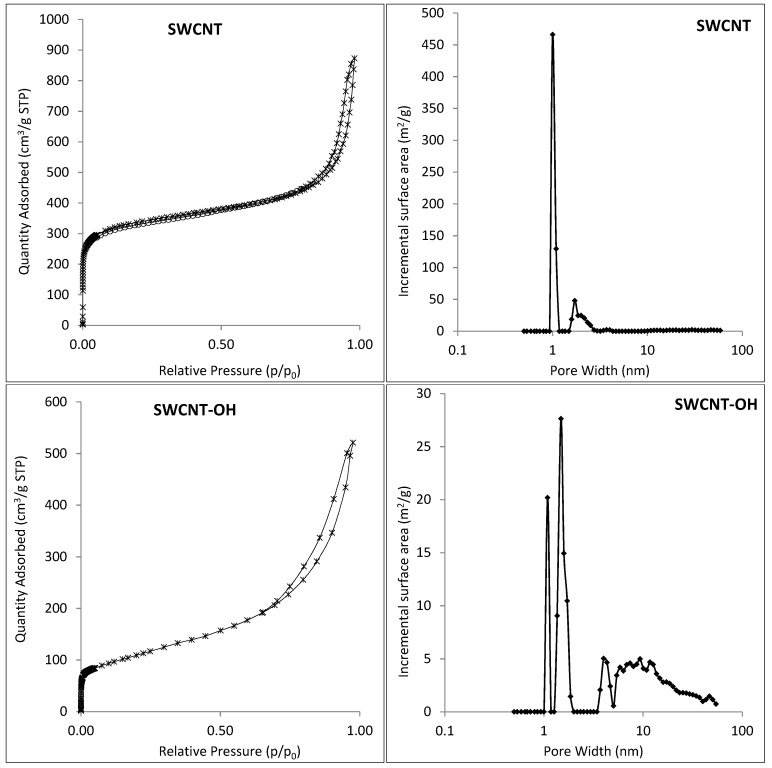

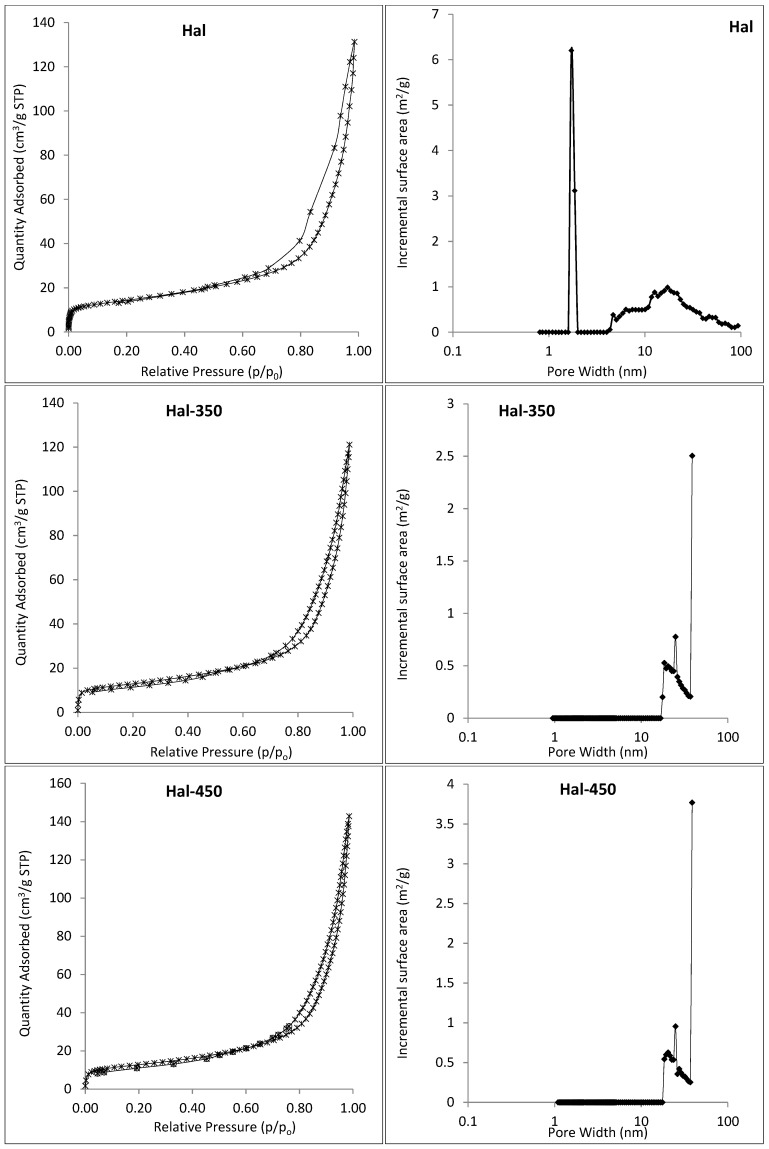

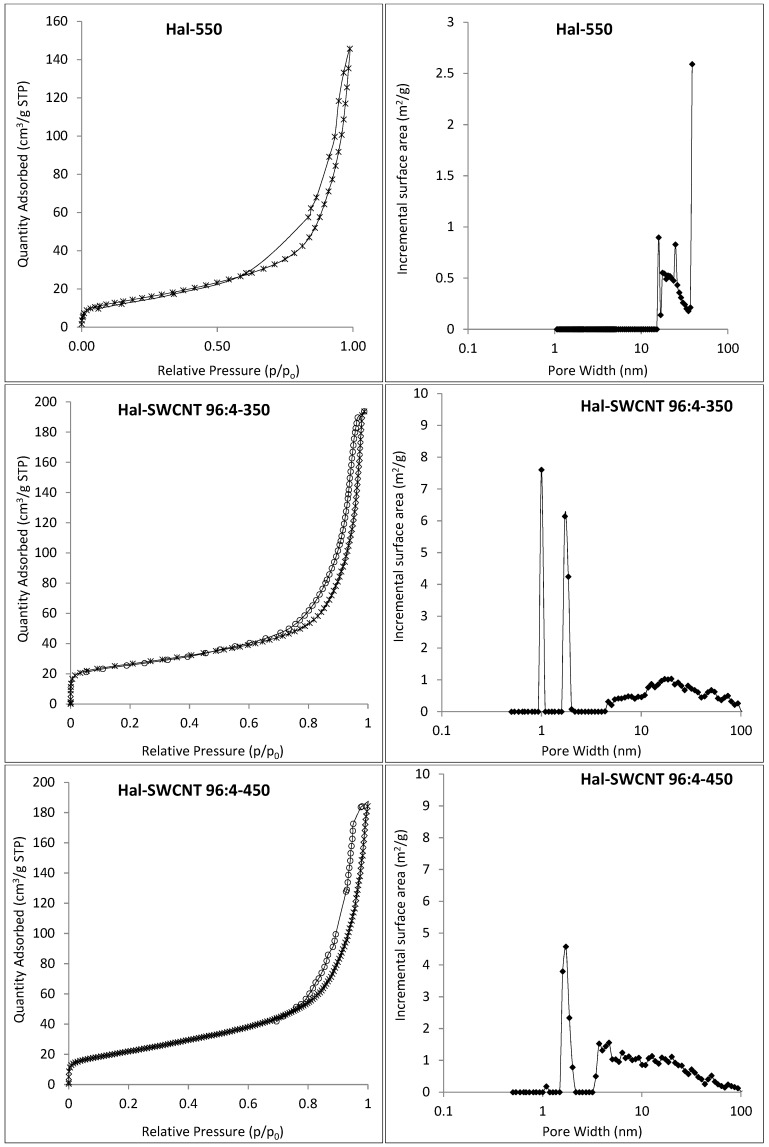

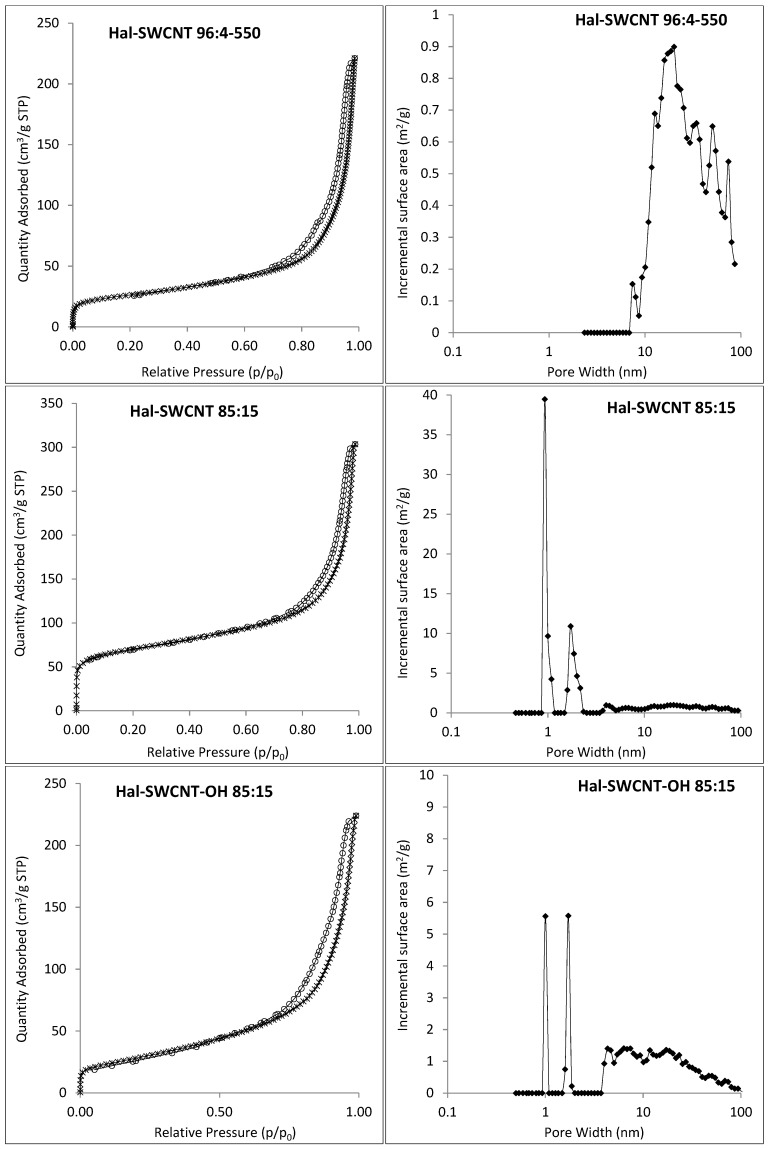

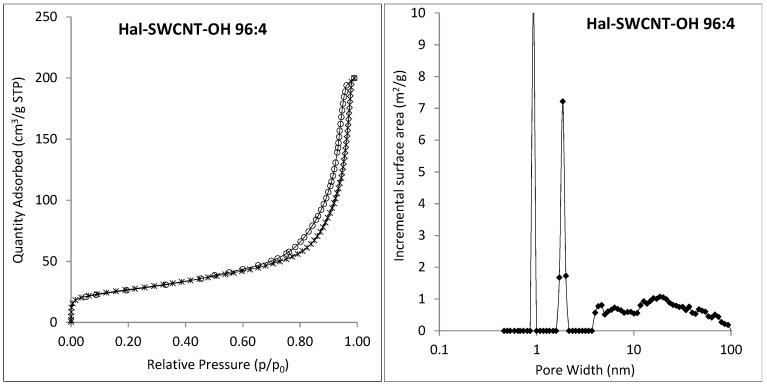
Nitrogen sorption-desorption isotherms (**left**) and pore size distribution (**right**) of the Hal, SWCNT, SWCNT-OH, Hal-350, Hal-450, Hal-550 and Hal-CNT composites.

Figure 5 presents the nitrogen sorption-desorption isotherms and pore size distribution of raw materials (halloysite, SWCNT, SWCNT-OH), thermally treated halloysite (Hal-350, Hal-450 and Hal-550 °C) and Hal-CNT composites. According to the IUPAC (International Union of Pure and Applied Chemistry) classification, all nitrogen adsorption-desorption isotherms showed IV type isotherms, which are representative for physical multilayer adsorption and capillary condensation [41]. For the IV isotherm type, the adsorption volume rapidly increases at low relative pressures of less than 0.01 due to the interaction of the adsorbate molecules with the higher energetic region, followed by the interaction with the less energetic region. When the monolayer formation of the adsorbed molecules is complete, multilayer formation starts to take place corresponding to the sharp knee of the isotherms. As the relative pressure approaches 1, an abrupt rise indicates the bulk condensation of gaseous nitrogen to liquid. The shape of hysteresis loops (classified as H3) indicates that the sorbent nature was aggregated of platelike particles forming a slit-like structure (can be seen in Figure 2 for Hal-SWCNT 96:4-350), which was related to non-rigidity of the adsorbent [42,43]. In Figure 5 (right), pore size distribution of all samples was depicted. SWCNT consisted mainly of micropores, while SWCNT-OH contained both micro and mesopores. Raw Hal exhibited a diverse structure including the highest fraction of micropores (0–2 nm), and the average fraction of mesopores (2–50 nm) and the lowest fraction of macropores (>50 nm). A much different structure was observed for thermally treated halloysite. As it was independent of the temperature, the Hal-350, Hal-450 and Hal-550 revealed only mesopores (15–39 nm). Heating changed the halloysite structure because a temperature rise induces a multistage dehydration of halloysite starting from water elimination from the surface (50–120 °C), then dehydration of structural water (200–400 °C) and then hydroxyl dehydration of the inner layer (400–550 °C). It results in the increase of the inner diameter of halloysite and the formation of thinner walls, giving a more porous and expanded structure [44]. 

The structure of Hal-CNT composites was closely related to the structure of original raw material. If Hal-SWCNT 85:15 and Hal-SWCNT 96:4-550 is excluded, the structure of the other Hal-CNT composites exhibited almost identical pore size distribution as raw Hal and SWCNT-OH. Interestingly, the sample Hal-SWCNT 85:15 was dominated by micropores, which isthe same as highly microporous SWCNT. In the sample treated at the highest temperature (Hal-SWCNT 96:4-550), mesopores and macropores were mainly revealed. This can be explained by high temperatures that destroyed aluminosilicates resulting in the collapse of micropores, which corresponds with abundant pores [45].

The BET SSA and pore structure properties are summarized in Table 4. Comparing the BET surface area of raw halloysite and heated halloysite samples, it is clear that thermal treatment did not affect the SSA greatly. Only the sample heated at 550 °C had a little higher SSA than raw halloysite. It is related to the hydroxyl dehydration of halloysite’s inner layer which occurs at 400–550 °C and causes the high mass loss of halloysite and transformation of the crystal structure of halloysite into an amphoric one. Similarly, Yu et al. [44] reported that when the calcination temperature rises (25–550 °C), the average pore size of halloysite increased from 18.1 nm to 29.8 nm, but the same BET surface area increased only marginally. From approximately the twice lower value of volume of pores for Hal-350, Hal-450, Hal-550 comparing to raw Hal, the authors assumed that they exhibited poor adsorption properties. The benefit from heating is the creation of the structure of halloysite being more open and then suitable to be based material for accommodation of carbon nanotubes.

The BET surface area and pore volume of all Hal-CNT composites were much larger than both raw and calicined halloysite, suggesting that only the adding of carbon nanotubes had a significant activation effect on SSA. Both the type and amount of CNTs affected the BET surface area of Hal-CNT composites. The sample with 15% loading of SWCNT (Hal-SWCNT 85:15) had over twice the BET surface area and a larger fraction of micropores than the sample with 4% SWCNT loading. Similarly, the BET surface area of Hal-SWCNT-OH 85:15 was higher than Hal-SWCNT-OH 96:4. It was also found that initial properties of raw carbon nanotubes also affected the BET surface area of halloysite-CNT composite. The sample Hal-SWCNT 85:15 had more than two times larger BET surface area than Hal-SWCNT-OH 85:15. It can be explained by more microstructural properties and a much larger initial SSA of raw SWCNT than SWCNT-OH.

Cheng et al. [46] observed a gentle decrease in the BET surface area of TiO_2_/SiO_2_ composite when the calcination temperature increased from 200 to 600 °C. Similarly, Sun [47] reported that the increase in the calcination temperature from 300 °C to 700 °C reduced the BET surface area of TiO_2_-zeolite composite from 80 m^2^/g to 22 m^2^/g. The finding with the most favorable temperature to calcine the sample is important, due to the energy input which created the expanded halloysite structure able to accommodate carbon nanotubes. The temperature of 350 °C was found to be the most optimum because it yielded the same mesoporosity of Hal-350 as Hal-450 and Hal-550, although the sample heated at 550 °C showed slightly higher SSA. In addition, microporosity/mesoporosity and the BET surface area of Hal-SWCNT 96:4-350 were very similar to Hal-SWCNT 96:4-450, but under a lower energy input. Then, the higher calcination temperature (550 °C) reduced the SSA of Hal-SWCNT 96:4-550 to 80 m^2^/g.

### 3.2. Adsorption Isotherms

**Figure 6:**
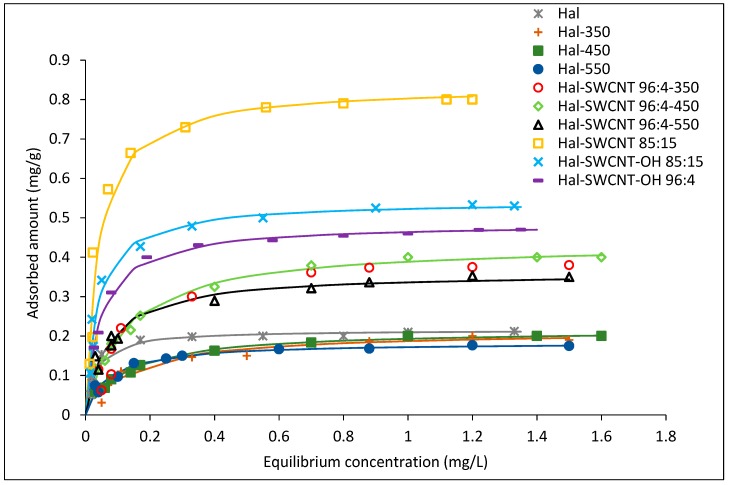
Langmuir adsorption isotherms of anthracene on Hal, Hal-350, Hal-450, Hal-550 and Hal-CNT composites. The solid line represents the Langmuir fitting.

Figure 6 shows the adsorption isotherms of ANT on Hal-CNT composites. According to the Giles classification, adsorption isotherms for all sorbents correspond to the L1 type. Asymptotic plateau suggests that sorbent has a limited adsorption capacity. It means that when more sites in the sorbent are filled, it becomes gradually impossible for a bombarding particle to occupy the available adsorption sites [48,49].

The experimental data were fitted to the Freundlich and Langmuir models, according to Equations (2) and (3) respectively [35]:

(2) $$Q_{e}=K_{f}C_{e}^{1/n}$$

(3) $$Q_{e}=\frac{a_{L}bC_{e}}{1+bC_{e}}$$

where: $Q_{e}$ is the equilibrium amount of absorbed ANT in mg per gram of sorbent (mg/g), $K_{f}$ is the Freundlich adsorption coefficient ((mg/g)(L/mg)*^n^*), $C_{e}$ is the equilibrium concentration (mg/L), n is a number describing the surface heterogeneity and sorption intensity, $a_{L}$ is the maximum adsorption capacity (mg/g), and b is the Langmuir fitting parameter (L/mg). A trial and error procedure was used for the non-linear method using the solver add-in with a Microsoft Excel spreadsheet. The equation parameters were obtained by means of minimization of the sum of squared errors (SSE) and listed in Table 5. Based on the R^2^ value, it was found that the Langmuir model better describes the course of adsorption. From the shape of the isotherms, it is evident that the Hal-SWCNT 85:15 exhibited the greatest sorption capacity, while Hal-SWCNT 96:4-550 the lowest. This is also confirmed by the maximum adsorption ($a_{L}$) and value of the $K_{f}$ constant which were both the highest for Hal-SWCNT 85:15 and the lowest for Hal-SWCNT 96:4-550.

From isotherms in Figure 6 and parameters in Table 5, it is clear that raw halloysite and Hal-350, Hal-450 and Hal-550 exhibited very low adsorption capacity of ANT as an effect of low SSA and dominant mesoporosity unable to uptake organic micro-pollutants. This brings very important issue that halloysite itself is useless as an adsorbent of organic micro-pollutants. 

**Figure 7:**
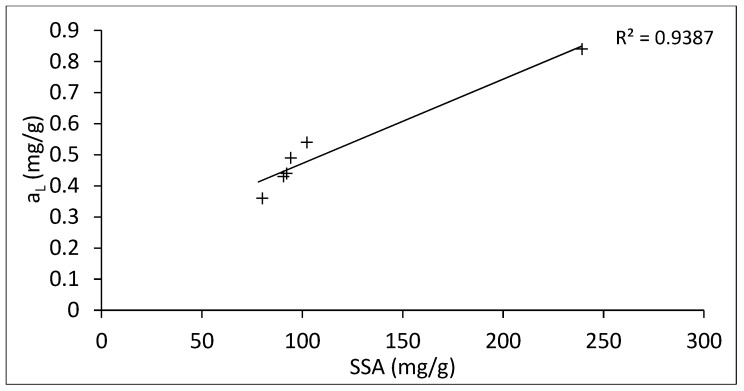
The maximum absorbed amount ($a_{L}$) of ANT as a function of BET SSA of Hal-CNT composites. The solid line represents linear fitting.

In Figure 7, the maximum absorbed amount ($a_{L}$) of ANT was plotted along with SSA of Hal-CNT composites. It is clear that there is a linear relation between SSA of sorbents and absorbed amount of ANT. A similar correlation between maximum adsorption capacity and SSA was found for activated carbon [50]. On the other hand, the adsorption capacity can be also affected by microporous characteristics of sorbents, surface functional groups or in case of carbon nanotubes, by their size and the number of graphene sheets [34]. 

Overall, these results show that the highest adsorption potential was observed for Hal-SWCNT 85:15. Comparing adsorption capacity and SSA for samples Hal-SWCNT 85:15 and Hal-SWCNT-OH 85:15, it can be seen that samples with SWCNT-OH have over twice the lower value of SSA and 1.5 times lower adsorption capacity of ANT. Then, when samples were contrasted with the same type of carbon nanotubes but with different amounts (i.e., Hal-SWCNT 85:15 and Hal-SWCNT 96:4-450), it can be observed that both SSA and adsorption capacity are definitely higher for samples with higher amounts of carbon nanotubes. This suggests that both the type and amount of carbon nanotubes play an important role in the adsorption potential of Hal-CNT composites. Other factors like porous structure and functional groups seem to be, in that case, negligible.

### 3.3. Adsorption Kinetics 

**Figure 8:**
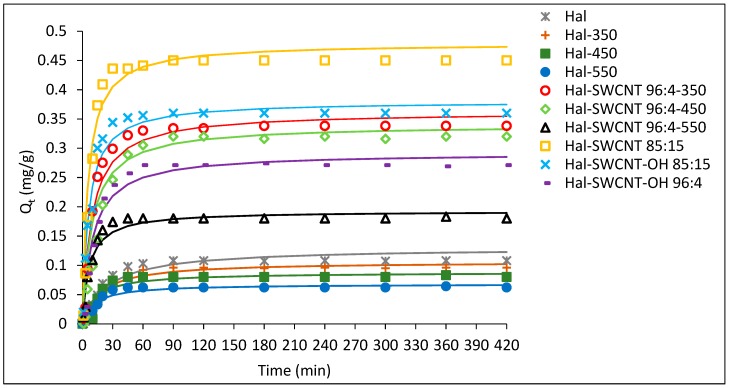
Adsorption kinetic curves of ANT on Hal, Hal-350, Hal-450, Hal-550 and Hal-CNT composites.

The sorption kinetic curves of ANT on Hal, Hal-350, Hal-450, Hal-550 and Hal-CNT composites are depicted in Figure 8. The kinetic equilibrium was reached faster (35–45 min) for the samples Hal-SWCNT 85:15, Hal-SWCNT-OH 85:15 and Hal-SWCNT 96:4-550 than for other samples (60–90 min). In case of samples with Hal:CNT ratio, 85:15 it is a direct consequence of the higher amount of carbon nanotubes that adsorb solute molecules very fast. Many authors proved that carbon nanotubes are able to uptake adsorbates faster than any other common adsorbent [33,51,52]. This is because of the external surface of carbon nanotubes, which is available for sorption and guarantees a quick uptake. Contrary, when the amount of carbon nanotubes is lower, adsorption occurs mainly in the microporous structure of halloysite, resulting from dehydration during heating, where the molecules need time to access the microporosity through the porous network.

The experimental data were fitted using pseudo first kinetic order and pseudo second kinetic equations expressed as follows, respectively [34]:

(4) $$ln\frac{\left(Q_{e}-Q_{t}\right)}{Q_{e}}=-K_{1}t$$

(5) $$\frac{t}{Q_{t}}=\frac{1}{K_{2}{\left(Q_{e}\right)}^{2}}+\frac{t}{Q_{e}}$$

where: $Q_{e}$ and $Q_{t}$ are the amount of ANT adsorbed at equilibrium and at time (t), $K_{1}$, $K_{2},$ are the pseudo-first-order, pseudo-second-order constants respectively. Furthermore, based on the pseudo-second-order model, the half adsorption time (*t*_1/2_) (Equation (6)) and the initial adsorption rate (*h*) were calculated (Equation (7)) [53].

(6) $$t_{1/2}=\frac{1}{K_{2}*Q_{e}}$$

(7) $$h=K_{2}Q_{e}^{2}$$

The calculated parameters are presented in Table 6. The pseudo-first-order model was not suitable for describing the experimental data, especially at the short contact time. It was evidenced by the low value of the approximation (<0.9). However, the pseudo-second-order model described the adsorption of ANT on the studied samples (excluding Hal-450) very well. The calculated ($Q_{t\left(cal\right)}$) values corresponded well with experimental data ($Q_{t\left(exp\right)}$). This suggests that the adsorption of ANT obeyed the second-order kinetics and this model could be used to determine kinetic parameters. Based on these parameters, it was found that the adsorption kinetics were the slowest for halloysite samples (raw and heated). Then, the values of parameters $t_{1/2}$, h, $K_{2}$ were almost twice as high for Hal-SWCNT 85:15 and Hal-SWCNT-OH 85:15 than for the other composites, suggesting a positive correlation between the amount of carbon nanotubes and the adsorption rate. This large difference is related to available adsorption sites on the external surface of carbon nanotubes that are in direct contact with the adsorbate molecules. Thus, composites containing higher amounts of carbon nanotubes had more external surface and were able to adsorb anthracene quicker than the composite with a low amount of CNT. Similarly, Strachowski et al. [54] found that adsorption of phenolic compounds was two times faster by carbon nanotubes than activated carbon due to the morphology of carbon nanotubes allowing adsorption on their outer surface. The type of carbon nanotubes (SWCNT or SWCNT-OH) did not greatly affect the adsorption rate because the constant $K_{2},$ half-adsorption time and adsorption rate for the Hal-SWCNT 85:15 and Hal-SWCNT-OH 85:15 are described by similar values. Interestingly, values of $t_{1/2}$, h and $K_{2}$ for Hal-SWCNT 96:4-550 suggest very fast adsorption rates, however the amount of ANT adsorbed at equilibrium was low. It can be related with macroporosity of this sample. From Figure 5 and Table 4, it is clear that Hal-SWCNT 96:4-550 contains mainly mesopores and macropores with the SSA value of 80 mg/m^2^. In this case, ANT molecules were adsorbed only on the outer surface of SWCNT or Hal that were directly accessible because the mesoporous and macroporous structure is empty of adsorption sites. Basically, they play important role in the transport of adsorbate to microporosity, but not in proper adsorption [55].

### 3.4. Adsorption Rate-Controlling Mechanisms 

The adsorption of adsorbates from the aqueous solution under shaking conditions include two stages: (1) Diffusion of adsorbate molecules from the liquid phase to the surrounding of the surface of sorbents; and (2) intraparticle diffusion of molecules deeply into the porous network of sorbents [56]. In order to evaluate the rate controlling mechanism, experimental data were fitted using Weber and Morris intraparticle diffusion model expressed by the following equation [57]:

(8) $$\frac{Q_{t}}{Q_{e}t}=k_{D}t^{-0.5}+I$$

where: $k_{D}$ is the rate constant for intraparticle diffusion, I is an indicator of the thickness of the boundary layer. 

**Figure 9:**
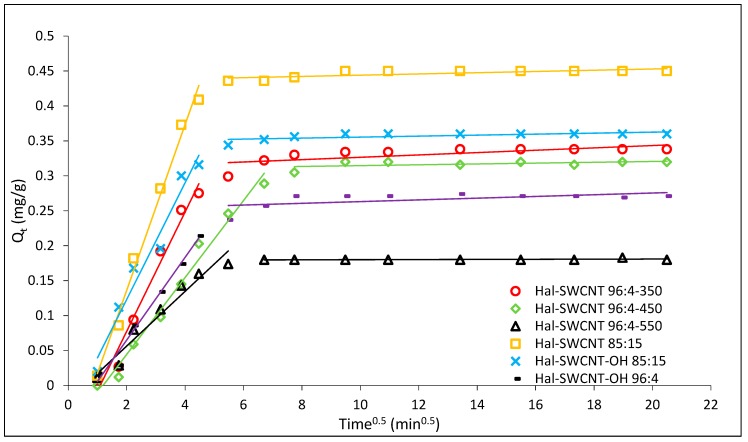
Interparticle diffusion plots for adsorption of ANT on Hal-CNT composites.

Figure 9 shows the adsorbed amount of ANT versus the square root of the contact time. If only the intraparticle diffusion limits adsorption rate, the $Q_{t}$ versus $t^{0.5}$ plot is linear and passes through the origin. The curve in Figure 9 is multilinear and splits into two straight lines suggesting that the rate of adsorption of ANT is limited by at least two steps [58]. The first linear part, from 1 to 45/60 min, is controlled by film diffusion or intraparticle diffusion or both. The second line, from 60–420 min, showed a very low slope suggesting the establishment of equilibrium. It corresponds to slowing down of intraparticle diffusion due to a low concentration of ANT in the solution. Therefore, the final adsorption is rather controlled by film diffusion [53].

The interparticle diffusion rate and thickness of the boundary layer for the first step of adsorption are presented in Table 7. The lowest $k_{D}$ value was obtained for Hal-SWCNT 96:4-550 while the highest for Hal-SWCNT 85:15 and Hal-SWCNT 96:4-450. It suggests that the effect of interparticle diffusion depends on microporosity of the sample. Therefore, intraparticle diffusion played a more important role in the adsorption of ANT on microporous samples than on a macroporous one. In case of a macroporous sample, the film diffusion was a dominant factor limiting the rate of ANT adsorption. 

From the value I, it is clear that the film diffusion is larger for composites with higher amounts of carbon nanotubes, probably due to a more intensive boundary layer effect of ANT onto their external surface [59]. This brings two important things: (1) An increase in the amount of carbon nanotubes in Hal-CNT composites greatly affects adsorption mechanism; (2) In the case of microporous composite film, diffusion seems to be equally important to control adsorption as intraparticle diffusion. It can be explained by the morphological characteristic of microporous Hal-SWCNT 85:15. They have adsorption sites both on their external surface and also within the microporosity of the sample [33]. 

## 4. Conclusions

Overall, this study demonstrates that halloysite-carbon nanotubes composites can be regarded as a novel sorbent for the removal of ANT (an emerging organic micro-pollutant) from water matrices. It is because of their higher BET surface area, higher adsorption capacity and faster adsorption kinetics compared to raw, as well as heated halloysite. The thermal treatment makes the halloysite structure more expanded and suitable to be based material for carbon nanotubes. The use of carbon nanotubes with high specific surface area (SWCNT) was a crucial factor to enhance the adsorption potential of Hal-CNT granules. The use of SWCNT-OH seems to be less worth in terms to adsorption capacity for anthracene, however, they could be more attractive for dissociated forms of other pollutants due to the interaction between their functional groups and OH from SWCNT-OH. Another key factor was the amount of carbon nanotubes. Due to the increase in the amount of carbon nanotubes, the higher BET surface area, the higher adsorption capacity and the higher adsorption rate of Hal-CNT composites, were noted. It is explained by the superb adsorption properties of raw carbon nanotubes.

Significantly, this study shows that even small amounts of carbon nanotubes are enough to enhance the adsorption potential of Hal-CNT composite in terms of the adsorption rate and capacity. This study can be a starting point for the implementation of carbon nanotubes as currently their wider usage is limited by high cost. Hal-CNT composites can be employed to remove organic micro-pollutants from drinking water sources, WWTP effluent or industrial streams. Also, their granulated form is prone to regeneration which aids their use in large-scale systems, both in batch and continuous flow conditions. 

## Figures and Tables

**Table 1:** Properties of carbon nanotubes.

Name	Outer Diameter ^1^ (nm)	Length ^1^ (µm)	Purity ^1^ (wt%)	OH Content ^1^ (%)
SWCNT	<2	5–30	95	not applicable
SWCNT-OH	<2	5–30	90	3.7

^1^ Data provided by manufacturer.

**Table 2:** Properties of anthracene.

Compound	Chemical Structure	Molecular Mass ^1^ (g/mol)	log K_ow_^1^ (-)	Solubility in Water 20 °C ^1^ (mg/L)
ANT	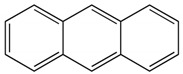	178.55	4.45	0.043

^1^ data taken from https://pubchem.ncbi.nlm.nih.gov/.

**Table 3:** Types of the thermally treated samples and their preparation conditions.

Symbol	Carbon Nanotubes Type	Halloysite to CNT Weight Ratio (%)	Calcination Temperature (°C)
Hal-350	-	-	350
Hal-450	-	-	450
Hal-550	-	-	550
Hal-SWCNT 96:4-350	SWCNT	96:4	350
Hal-SWCNT 96:4-450	SWCNT	96:4	450
Hal-SWCNT 96:4-550	SWCNT	96:4	550
Hal-SWCNT 85:15	SWCNT	85:15	450
Hal-SWCNT-OH 85:15	SWCNT-OH	85:15	450
Hal-SWCNT-OH 96:4	SWCNT-OH	96:4	450

**Table 4:** The characteristic of SWCNT, SWCNT-OH, Hal, Hal-350, Hal-450, Hal-550 and Hal-CNT composites from nitrogen sorption-desorption measurements.

Adsorbent	BET Specific Surface Area (m^2^/g)	Total Volume in Pores (cm^3^/g)	Total Area in Pores (m^2^/g)
SWCNT	1124.6	0.88	945.6
SWCNT-OH	410.63	0.6	257.52
Hal	50.36	0.19	32.69
Hal-350	45.15	0.06	45.86
Hal-450	44.89	0.08	50.18
Hal-550	54.16	0.07	56.55
Hal-SWCNT 96:4-350	90.56	0.25	55.64
Hal-SWCNT 96:4-450	92.15	0.23	54.39
Hal-SWCNT 96:4-550	80.09	0.27	47.24
Hal-SWCNT 85:15	239.22	0.33	153.12
Hal-SWCNT-OH 85:15	102.24	0.29	68.28
Hal-SWCNT-OH 96:4	94.20	0.26	62.12

**Table 5:** The parameters of the Langmuir and Freundlich equations and correlation coefficients for the adsorption of anthracene on Hal, Hal-350, Hal-450, Hal-550 and Hal-CNT composites.

Sample	Langmuir			Freundlich		
	$a_{L}$ (mg/g)	b (L/mg)	R^2^ (-)	$K_{f}$ ((mg/g)·(L/mg)^*n*^)	n (-)	R^2^ (-)
Hal	0.21	32.30	0.9592	0.21	5.01	0.8171
Hal-350	0.21	7.21	0.9548	0.19	3.05	0.9045
Hal-450	0.22	8.32	0.9871	0.19	3.52	0.9518
Hal-550	0.18	14.47	0.9633	0.17	4.51	0.8805
Hal-SWCNT 96:4-350	0.43	6.67	0.9531	0.37	2.92	0.8905
Hal-SWCNT 96:4-450	0.44	8.24	0.9867	0.38	3.52	0.9464
Hal-SWCNT 96:4-550	0.36	13.84	0.9609	0.34	4.07	0.9596
Hal-SWCNT 85:15	0.84	24.78	0.9519	0.84	4.38	0.8174
Hal-SWCNT-OH 85:15	0.54	26.29	0.9646	0.54	4.53	0.8701
Hal-SWCNT-OH 96:4	0.49	20.87	0.9963	0.47	5.06	0.8819

**Table 6:** The parameters of the pseudo-second-order kinetic equation for the adsorption of ANT on Hal, Hal-350, Hal-450, Hal-550 and Hal-CNT composites.

Sample	Pseudo-Second-Order Equation Parameters					
	$K_{2}$ (g/(mg·min))	$Q_{t\left(exp\right)}$ (mg/g)	$Q_{t\left(cal\right)}$ (mg/g)	$t_{1/2}$ (min)	h (mg/(g·min))	R^2^
Hal	0.23	0.108	0.11	18.27	0.006	0.9438
Hal-350	0.2	0.096	0.1	17.61	0.006	0.9686
Hal-450	0.088	0.083	0.09	16.27	0.006	0.870
Hal-550	0.268	0.06	0.055	8.76	0.007	0.9498
Hal-SWCNT 96:4-350	0.296	0.35	0.36	9.33	0.039	0.964
Hal-SWCNT 96:4-450	0.31	0.32	0.34	9.49	0.036	0.972
Hal-SWCNT 96:4-550	0.757	0.18	0.19	6.96	0.027	0.960
Hal-SWCNT 85:15	0.479	0.45	0.38	5.49	0.087	0.970
Hal-SWCNT-OH 85:15	0.428	0.36	0.38	6.16	0.062	0.967
Hal-SWCNT-OH 96:4	0.292	0.27	0.34	10.07	0.029	0.972

**Table 7:** Interparticle and film diffusion rate parameters for ANT adsorption on Hal-CNT composites.

Sample	$k_{D}$ (mg/g·min^0.5^)	I (mg/g)
Hal-SWCNT 96:4-350	0.013	0.143
Hal-SWCNT 96:4-450	0.015	0.09
Hal-SWCNT 96:4-550	0.006	0.12
Hal-SWCNT 85:15	0.015	0.229
Hal-SWCNT-OH 85:15	0.012	0.18
Hal-SWCNT-OH 96:4	0.01	0.113

## Supplementary Materials

The following are available online at https://www.mdpi.com/2079-4991/9/6/890/s1, Figure S1: Digital photograph of Hal-SWCNT 85:15, Figure S2: EDX results of Hal-CNT composites, Figure S3: ATR-FTIR spectra of: (a) SWCNT (b) SWCNT-OH.
