# Supplementary material for: Preparation, Characterization and Adsorption Potential of Grainy Halloysite-CNT Composites for Anthracene Removal from Aqueous Solution
Source: Nanomaterials (Basel). 2019 Jun 17;9(6):890. doi: 10.3390/nano9060890 (PMC6630252; doi:10.3390/nano9060890)
Supplement: Supplementary file 1 [file nanomaterials-09-00890-s001.pdf]

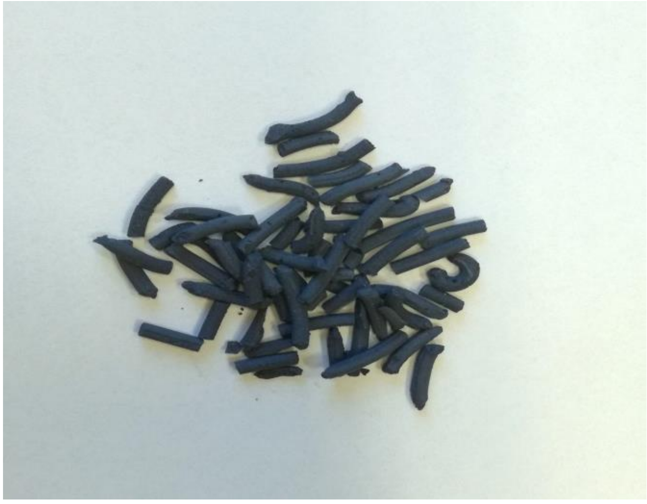

Figure S1. Digital photograph of Hal-SWCNT 85 15

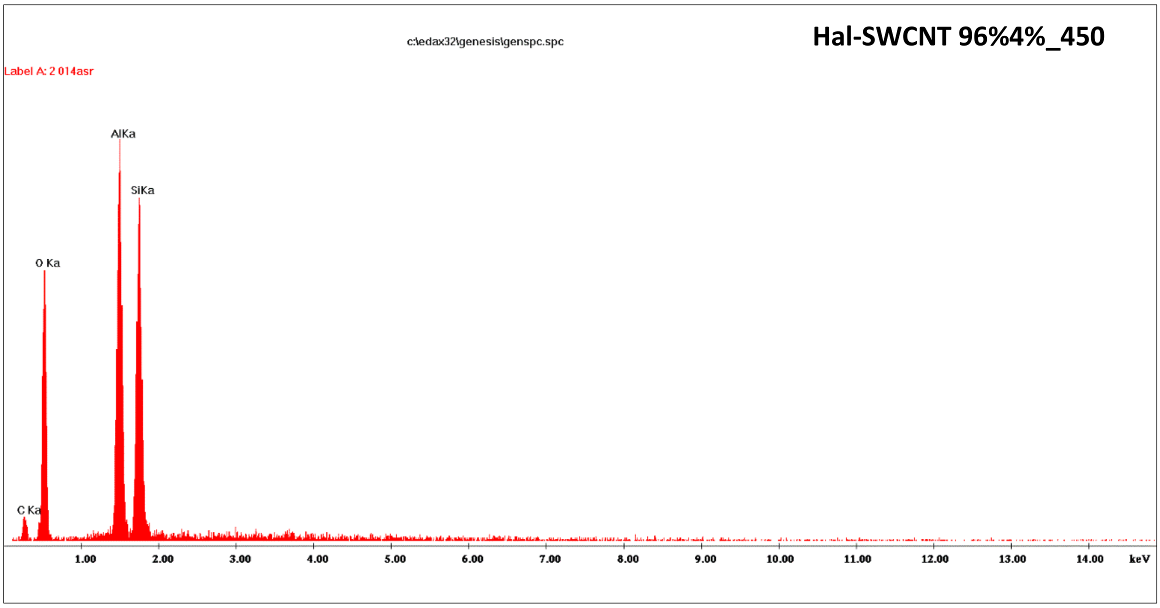

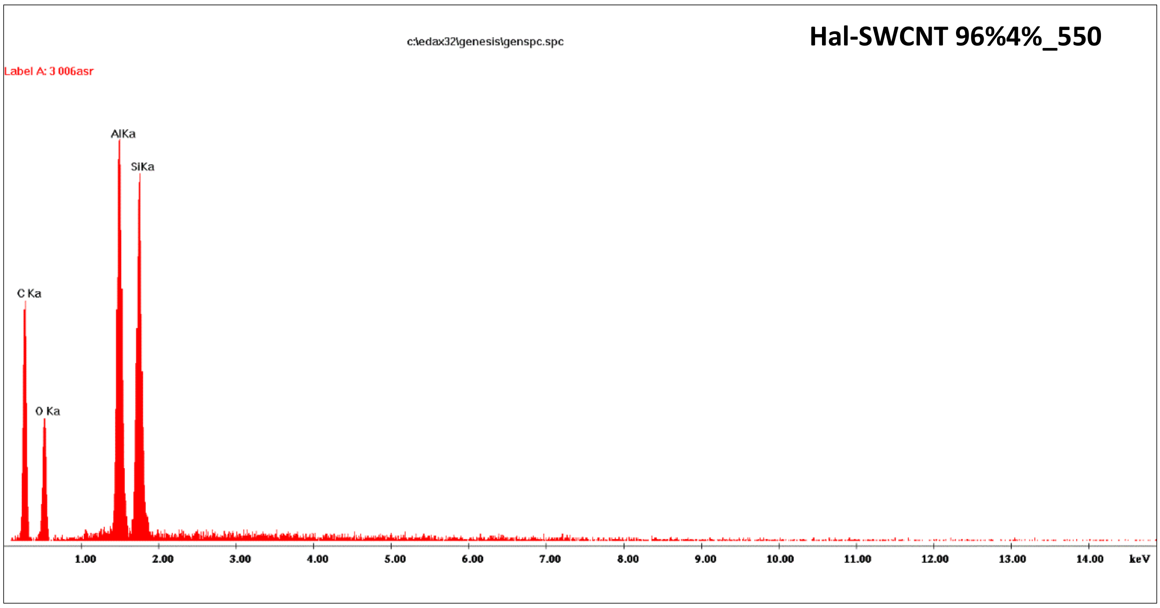

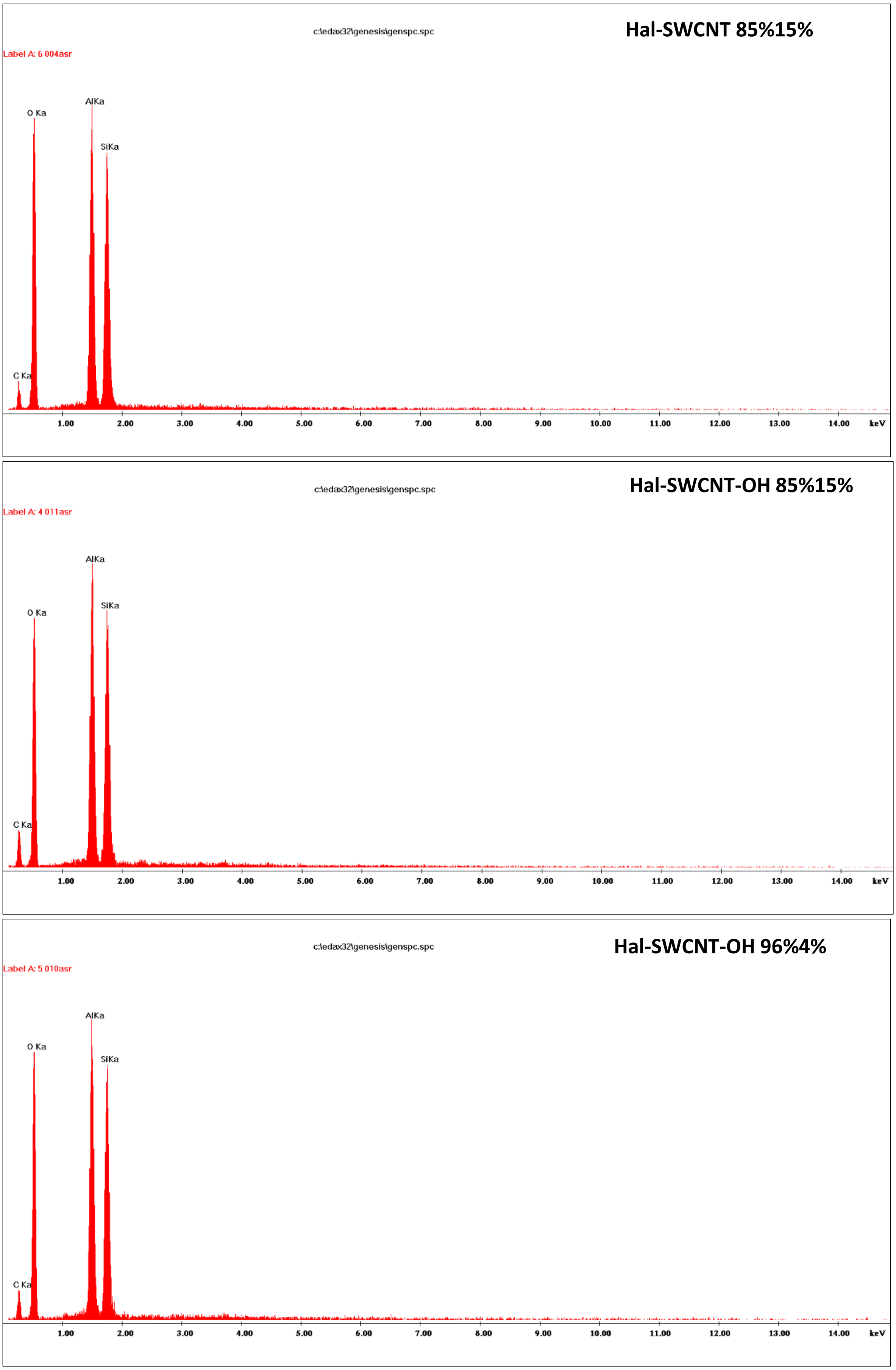

Figure S2. EDX results of Hal-CNT composites

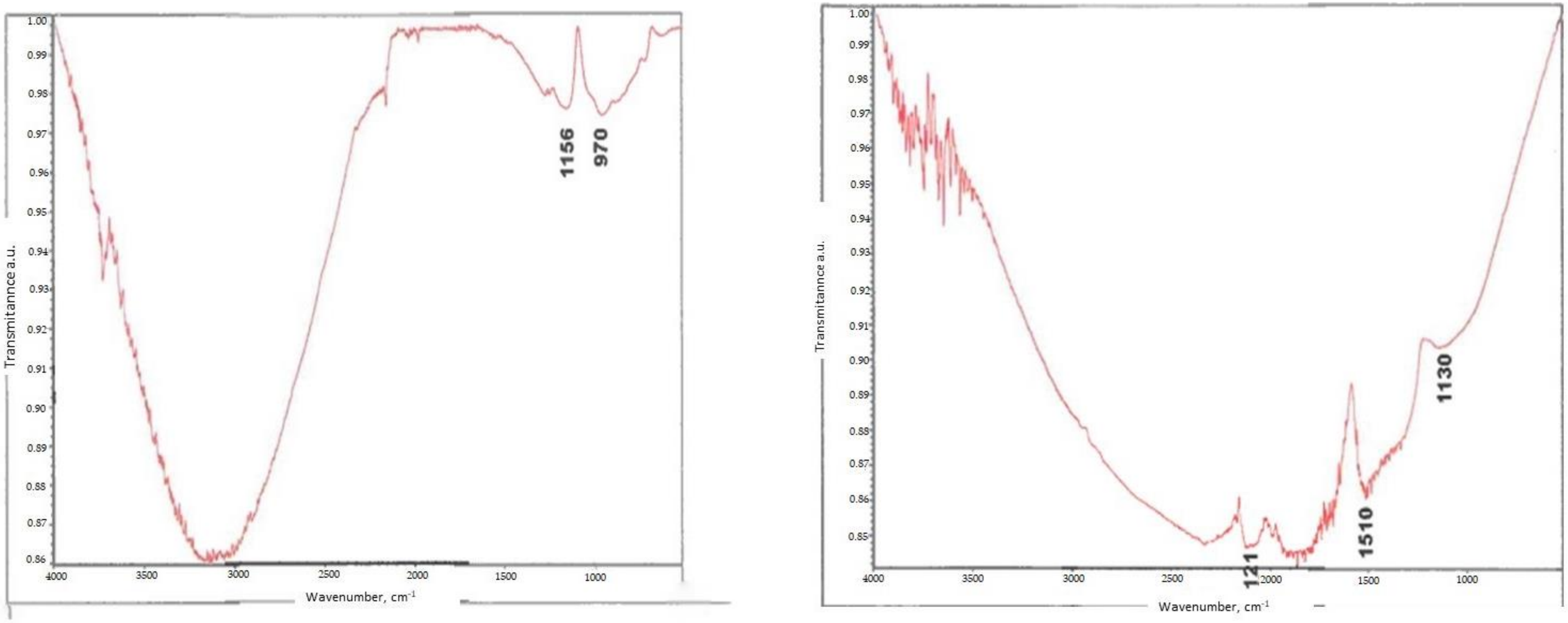

a) b)

Figure S3. ATR-FTIR spectra of: a) SWCNT b) SWCNT-OH.
